# ‘I'm ruined’: Young people's and their mothers' experiences of living with, and receiving a diagnosis of, borderline personality disorder: An interpretative phenomenological analysis

**DOI:** 10.1111/papt.12549

**Published:** 2024-10-07

**Authors:** L. Marriner, M. Larkin, G. Urquhart Law, S. Kaur Bhogal

**Affiliations:** ^1^ Centre for Applied Psychology School of Psychology, University of Birmingham and Birmingham and Solihull NHS Foundation Trust Birmingham UK; ^2^ Institute of Health and Neuroscience, Aston University Birmingham UK; ^3^ Centre for Applied Psychology School of Psychology, University of Birmingham Birmingham UK; ^4^ Mental Health Division Birmingham Women's and Children's Hospital Foundation NHS Trust Birmingham UK

**Keywords:** adolescence, borderline personality disorder, dyadic research, mental health, multi‐perspectival design, stigma

## Abstract

**Background:**

The adolescent borderline personality disorder (BPD) diagnosis has been widely debated for many years. Strikingly, young people's experiences of both receiving a diagnosis of BPD, and of living with BPD, are largely under‐explored. The current study seeks to address these gaps in the literature in a multi‐perspectival design utilising young people–parent dyads.

**Methods:**

Young people (aged 16–18 years) with a diagnosis of BPD (*n* = 5) and their mothers (*n* = 5) were recruited from two NHS Community Mental Health Services in the West Midlands and participated in semi‐structured interviews. Data were analysed using interpretative phenomenological analysis [IPA].

**Findings:**

Analysis identified two superordinate themes with five subthemes: (1) The “ugly” reality of living with BPD (providing a stark insight into what it is like to live with the unpredictable nature of experiences labelled as BPD), and (2) The diagnosis that dare not speak its name (highlighting the complexities of how the diagnosis itself is experienced by participants as a symbol of personal and permanent defectiveness and danger).

**Discussion:**

Findings highlight a clear commonality of experience centred around the intensity of the young people's emotional distress and the accompanying pressure on parents to keep young people safe, both of which services must strive to do more to contain. Ultimately, the costs of receiving a BPD diagnosis appear to outweigh the benefits, and this paper adds support to calls for change in respect to how we conceptualise difficulties labelled as BPD and how we communicate about these difficulties, in order to avoid causing harm.

## INTRODUCTION

Borderline personality disorder [BPD] is conceptualised as a severe mental health disorder defined by the Diagnostic and Statistical Manual of Mental Disorders [DSM‐V‐TR] as disturbances across at least five of nine domains. To reach the threshold for diagnosis, this disturbance has to persist for at least 1 year. The domains are: instability in emotion regulation, interpersonal relationships, impulse control and/or identity, self‐harm and suicidal behaviours, inappropriate intense anger, feelings of emptiness, efforts to avoid abandonment, and paranoid ideation. The DSM‐V‐TR allows for a diagnosis of BPD in young people according to the same criteria as in adults. BPD is the most commonly diagnosed PD in young people with prevalence rates ranging from 3% in the general population, 11% in outpatient mental health settings, 19%–53% in inpatient settings, and 78% in suicidal adolescents attending A&E (Guile et al., 2018).

Despite the legitimate inclusion of an adolescent BPD diagnosis in psychiatric nomenclature, the diagnosis is shrouded by controversy, and a longstanding debate surrounding its application is evident within relevant literature. From one perspective, the adolescent BPD diagnosis is seen as unreliable and invalid, and as a vehicle for causing harm via stigmatisation, acting as a barrier to relevant treatment, and blocking the development of better‐fit alternatives (Chanen & McCutcheon, 2008; Hartley et al., 2022; Lomani, 2022). Resistance towards the BPD diagnosis more generally is also becoming evident within public spheres, for example via Social Media outlets such as Twitter, where relevant hashtags (e.g. #HumanNotPD and #PDInTheBin) track calls for change (Hartley et al., 2022). Additional critiques note that clinical presentations of BPD can vary greatly (Cavelti et al., 2021), and the landscape of diagnosis for young people is often further confused by a reluctance around using the formal term, resulting in a range of qualifying terms being added such as ‘emerging/possible/traits’, despite the NICE guidelines not supporting the use of such terms (NICE, 2009).

Contrastingly, advocates of the concept highlight empirical evidence that supports the validity and reliability of an adolescent BPD diagnosis. They argue that controversy contributes to the stigmatisation that surrounds the diagnosis, in turn leaving clinicians reluctant to diagnose, and thus causing harm by preventing access to suitable treatment and halting efforts to improve treatments (Cannon & Gould, 2022; Chanen, Sharp, & Hoffman, 2017; McKenzie et al., 2022; Sharp, 2017).

Strikingly, an important gap exists within the field; the experiences of young people diagnosed with BPD remain largely under‐explored. Research conducted with adults diagnosed with BPD has begun to identify the polarised nature of people's experiences of the diagnosis. It may be experienced as loaded with prejudice and as a source of misunderstanding (Nehls, 1999), or as necessary for sense‐making and access to treatment (Shepherd et al., 2017). Nevertheless, this literature is also remarkably limited, and we might assume that there may be additional connotations for adolescents, who are developing a sense of self during a developmental stage where the social consequences of stigmatisation can be significant (Tomova et al., 2021).

Consequently, the current study aims to address this gap in the literature by exploring the following research questions:

- How do young people, and their parents, experience and make sense of living with BPD, and what impact has BPD had on their lives?
- How do young people, and their parents, experience and make sense of receiving a label of BPD, and what does this label mean to them?

An interpretative phenomenological analysis [IPA] approach was selected to guide the research design and analysis, as this approach is centred on a commitment to exploring how individuals make sense of their major life experiences. It is phenomenological and idiographic, in that it is concerned with exploring particular instances of lived experience and the significance that these experiences have within the context of individuals' lives (Smith, Flowers & Larkin, 2009).

Ecological systems theory, such as the original nested model proposed by Bronfenbrenner (1979), and the alternative networked model proposed by Neal and Neal (2013), have long recognised the importance of examining the intricate social relationships and interactions both within and between the different systems surrounding an individual, in order to fully understand the complex forces surrounding the phenomenon of interest, and in particular child development. In this sense, knowledge and understanding about the experience of living with BPD may be considered to be located not only within the individual who has a diagnosis of BPD (the focal individual), but also within the experiences and social interactions with other members of the different systems surrounding the individual, and their individual experiences are likely to interact and overlap.

In order to acknowledge this contextual view of experience and to capture some of this complexity, the current study has utilised a novel multi‐perspectival design and is the first study of its kind to explore the experience of living with BPD amongst young person–parent dyads (young people and their relative parents), providing an opportunity for the scope of this research to extend beyond the individual to the family microsystem surrounding the young people. Furthermore, it is hoped that this design decision will extend the reach and validity of findings, as important issues may be evidenced from more than one perspective.

IPA involves a double hermeneutic; both the participant and the researcher engage in active interpretation and sense‐making (Smith & Osborn, 2008). The first author has worked with young people and adults with BPD across both inpatient and outpatient settings and was often struck by the chaotic and painful qualities of their lives and the derogatory and unjust ways in which people with BPD are often spoken about and perceived by others. These experiences fuelled a passionate desire to advocate for this group, to give them a voice and to improve outcomes and services.

## METHODS

### Aims

This qualitative study, using IPA, aimed to explore how young people and their parents experience living with and receiving a diagnosis of BPD.

### Participants and setting

Participants were recruited from two different, geographically separate, NHS Child and Adolescent Mental Health Services (CAMHS) within the West Midlands, across two participant groups: Young people who had received a diagnosis of BPD and their parents. Eligibility criteria for the young people included being aged 14–18 years old, having BPD been first discussed at least 3 months prior to taking part, being fluent in the English language, being under the care of a mental health service, and being able to access crisis support, and not deemed to be in immediate crisis. Parent/carer eligibility criteria included being the parent/carer of a young person who met the above criteria. In order to maximise participation, it was not necessary for both a young person and their relevant parent/carer to participate; both or either one of them could choose to take part.

#### Sampling

A purposive sampling strategy was used, in which clinical staff identified young people who met the eligibility criteria from their caseloads and approached them to ask if they would like to hear more about the research study. Clinicians sought young people's consent to also approach their relevant parent/carer about the study.

#### Participants

In total, five young people and five parents took part in this research, including three young people–parent dyads. Sixty percent of the young people were female, and 80% were white British, 1 young person was of mixed ethnicity. Young people were aged between 16 and 18 years old. Exact ages were not captured for parents but ranged between 31 and 40 and 51 and 60 years old. All parents were biological mothers (3 to daughters; 2 to sons), and the age of their child with BPD ranged from 16 to 18.

### Data collection

Semi‐structured 1:1 interviews were conducted by the first author. As recommended by Smith, Flowers, and Larkin (2009), a pre‐prepared semi‐structured interview schedule was utilised. Knight et al. (2003) conducted an IPA study exploring experiences of living with schizophrenia, and due to the similar focus, this was used as a starting point to inform and guide the development of the current research schedule. In research supervision with the 2nd and 3rd authors (researchers experienced in qualitative research and IPA), questions were collaboratively developed across four sections, aimed specifically at addressing the current research aims (1) Personal experience and understanding of their/their child's mental health issues, (2) Social understanding of their/their child's diagnosis and how it is contextualised within their lives, (3) Reflections on the impact BPD has had on their lives, and (4) Personal experiences and impacts of stigma. The interview schedule was implemented in a flexible manner and the interviewer asked spontaneous or follow‐up questions where appropriate. All interviews were audio recorded on an encrypted Dictaphone and transcribed verbatim.

### Data analysis

The 6‐step procedure for IPA, as outlined by Smith, Flowers, and Larkin (2009), was utilised to analyse the data. Larkin et al. (2018) guidelines for using IPA in multi‐perspectival designs helped to inform the multi‐level nature of the analysis. Initial codes and potential themes and subthemes were devised by the first author. Codes and themes were then discussed in research supervision with the 2nd and 3rd researchers.

### Ethics

Ethical approval was granted by the Black Country Research Ethics Committee. All participants were provided with detailed information about the study and provided written informed consent to participate. As dyads were used, there was a potential risk of internal anonymity, in that participants may recognise comments made in their significant other's interview. This risk was communicated in the information sheets. In addition, all participants were offered the opportunity to view their interview transcript to identify any sections that they would not like to be used in the final research report if they wished. Four participants opted to do this and highlighted a small number of quotes for exclusion.

### Analysis

The data analysis generated two super‐ordinate themes: ‘*The “ugly” reality of living with BPD*’ and ‘*The diagnosis that dare not speak its name*’. A total of five subthemes were generated from the data. Table 1 provides an overview of the themes and subthemes, and indicates the participants who contributed to each theme. Young people's names have been capitalised and young people and parents who are related have been allocated pseudonyms starting with the same letter, to aid with recognition in subsequent quotes.

**TABLE 1:** Thematic map.

Subthemes	YP	Mothers
Superordinate theme 1: The “ugly” reality of living with BPD		
1.1: Living in fear of what might happen next	All	All
1.2: Keeping my child safe is a 24‐h job and we shouldn't have to do this alone	N/A	All
Superordinate theme 2: The diagnosis that dare not speak its name		
2.1: The elephant in the room	All	All
2.2: Providing insight but also bringing threat	All	All
2.3: Worrying that outsiders might judge and insiders might despair	All	All

#### The “ugly” reality of living with BPD

This theme explores the important issues that emerged for both young people and mothers in relation to what living with BPD is like for them, poignantly described by SARAH as an “ugly” reality.

All the young people identified an unpredictable, uncontrollable nature to living with BPD, and their interviews offered an interesting insight into the impact of this uncontrollability in terms of ‘***living in fear of what might happen next’***. SARAH and DENISE illustrate this:

Sarah “I think a lot of it is living in fear of what is going to happen […] I worry about when I go into my meltdowns, because actually when I am in them it's very much ‘I can't cope, I don't want to be here, I want to die, this is awful’ […] but it is very scary, because I don't know what's going to happen, I never know what I am going to do […] am I going to end up flipping out? […] it's just this massive rage bubble, […] it's that unknown, unpredictability, it scares me […]”.

Denise “It's just shut‐down, and I'll start crying, and then I'll start punching walls and hitting my head on stuff […] I'll just be in this kind of, almost like I'm not there, I just kind of blank out, sometimes I will self‐harm, sometimes I don't, there will be lots of screaming, and sometimes it's like it's never going to end and it's the worst feeling I've ever experienced […]”

Sarah and Denise highlight several important issues for the young people in our study. Firstly, they both struggle with feeling like they are not in control of their behaviour, and this uncontrollability appears to perhaps be the most frightening part of having BPD. Secondly, a sense of being disconnected and not present in these moments appears to exacerbate the uncontrollable nature of their behaviour. Finally, this lack of control makes them feel dangerous; they worry that they could *cause* danger to themselves and that they could be *in* danger as they are unsafe and vulnerable.

This subtheme was reflected in all the mothers' interviews. All mothers spoke about a constant fear related to the danger their children posed to themselves, for example, Hollie: *“I dread waking up in the mornings and going into his room to see if he's actually done anything”*. Phoebe further illustrates this: “*he's alive, and that's what matters, that's all that sort of matters […] the main thing is he's alive, he's here”*. The repetition in Phoebe's quote emphasises the stark reality of how suicide is viewed by these mothers as a clear and present danger. For Nicola, her child's suicide was seen to be not only possible but also probable: *“[worrying about suicide is] always there. I did say to [clinician], I said ‘if somebody phoned me to say she succeeded [in committing suicide] I wouldn't be shocked’.”*

Mothers also recognised that the unpredictable nature of their children's behaviour made them potentially dangerous to others, including their families. Nicola was open and explicit in speaking about the fear that this unpredictability cultivated in her:

Nicola “At times, oh yeah [I am scared of her]. I've often joked to [clinician] ‘You know them films where the child is next to the bed with a knife’, I think sometimes, you just don't know. She will sometimes threaten you with it. She will say ‘When you're in bed tonight I'm going to kill you all’.”

The way Nicola frames her fear as a joke highlights the shame and taboo associated with expressing such concerns.

As a result of ***‘living in fear of what might happen next’***, ordinary family roles appeared to shift, and a reciprocal subtheme emerged in the mothers' interviews: **‘*keeping my child safe is a 24‐hour job and we shouldn't have to do this alone.’*** Debbie illustrates this:

Debbie “So some days are really sort of tough […] On those days I don't leave [Denise], I don't let her leave my side, I won't go out without her or let her be here on her own, because I know that can lead to self‐harm and other things, so I do try to spend those days particularly with her. I mean we are together a lot, but those days particularly I won't leave her on her own, or I will call my husband and get him to come back. […] So, it has had a large impact, I don't do anything without thinking about her, it's like having a baby […] it's like we've reverted back to that time in our lives.”

Here, Debbie adopts a role of vigilant new parent, with her child requiring constant surveillance to keep her alive. To maintain this role Debbie's entire life requires careful consideration and forward planning. The mothers reported feeling that they were on high alert and that they must be vigilant at all times. Hollie further highlights the active monitoring that is required:

Hollie “I do normally hear if he's awake in the night, I can hear his bed creaking, or hear him get up to go to the toilet. I always listen to how long he's in the bathroom for because one day, I thought ‘he's been in there too long’, I went in and he had broken a razor blade open and was self‐harming with that. So now we lock all the razors, razor blades away and have to lock all the medication away and hide the bleach. […] At one point he didn't have a door [on his bedroom] for months. I took his door off because he kept tying his telephone charger round his neck and round the door handle, and sitting in front of the door so we couldn't answer the door […] After a bit I put the door back on but I took the door handle off, so he still couldn't do anything, now he's got the door back to normal. None of the doors have locks on, anyway, even the bathroom doors. So we can get in. It's just if he pushes something up against the door, that's what I worry about.”

Hollie's alertness and preoccupation with minimising the risks present within the family home reminded the researcher of staff on acute inpatient wards, and managing this level of risk is clearly overwhelming. The stresses associated with managing this level of risk appeared to be exacerbated by mothers' perception that family systems were managing this without support from services, or as Hollie puts it ‘*I suppose I did sort of feel in it by myself in a way*.’. Katherine illustrates this point when speaking about the support she received from mental health services:

Katherine “Well I think there isn’t as much support[…] when they do discharge people from the hospital[…] there isn’t the support there would be, if you couldn’t walk you would get loads more support than you do because of the fact that they can’t be left alone because they self‐harm[…] We have been through periods where she couldn’t be left alone, and we were both holding down full time jobs, and it’s just impossible, and nobody cares really […] and what we felt as well was that actually what [mental health services] would have liked was if we had given up our jobs, because actually that would have then solved a bit of an issue for other people.”

In summary, all participants viewed the affective and behavioural aspects of BPD as unpredictable and uncontrollable and, consequently, they described living in constant fear of what might happen next. Mothers took on the responsibility of keeping their children safe and usual family life was significantly disrupted. This process appeared to be made more stressful by the perceived lack of support from mental health services in managing the constant risk. There was a clear sense of the intense, almost desperate, quality of the participants' lives.

#### ‘The diagnosis that dare not speak its name’

This theme addresses the stark avoidance and secrecy that appeared to surround the BPD diagnosis in the lives of the participants and offers some insights into the factors that may contribute to that caution, illustrating how the adolescent BPD diagnosis remains ***‘the diagnosis that dare not speak its name’***, deliberately echoing Chanen and McCutcheon (2008) as little appears to have changed.

Often, the BPD diagnosis was first communicated via a letter devoid of any accompanying explanation and left for participants to make sense of alone. Strikingly, even when the BPD diagnosis was communicated in person, all participants experienced this as brief and inadequate, as demonstrated by KATIE ‘*[I was given] a little bit [of information about BPD], but not as much as I should have been’*. KATIE's use of the word ‘should’ illustrates that participants recognised that the process of coming to know about their diagnosis was lacking.

All young people and mothers opted to conduct their own private research to come to know about BPD, as opposed to asking their clinicians about this. In addition, all young people spoke of avoiding sharing their BPD diagnosis with others; situating the BPD diagnosis as the ‘***elephant in the room’*** that was off‐limits, and unvoiced by all.

When the young people did disclose their diagnosis to others, this tended to be forced, as their symptoms drew attention to the ‘elephant in the room’ and they could no longer conceal their secret. For example, SARAH'S self‐harm scars acted as a physical symbol of her BPD that incited curiosity, and for DENISE and JOE difficulties within their relationships reached a point where an explanation for their behaviours was felt to be necessary.

The subtheme ***‘Providing insight but also bringing threat’*** offers some insights into factors that may motivate the avoidance that surrounds the BPD diagnosis. On the one hand, participants highlighted the BPD diagnosis as useful to them, as it provided a context for understanding their (or their child's) behaviour and difficulties, which prior to receiving the diagnosis had been missing. DENISE and Debbie illustrate this:

Denise “I had a bit of a hard time at first […] I didn't know what [BPD] was, I looked it up, and then I started to see that this is very very accurate, but at first I hated it, and then it was more that I have somewhere where I like belong, and I'm not crazy, because it's happened to lots of people before […] so it kind of gave me a reason about why I do [certain behaviours], because I like to know why I do stuff.”

Debbie “[When I first heard the BPD diagnosis] I was upset […] but then I started to read up about it and I thought ‘do you know what, this diagnosis actually matches’ […] and I picked that up straight away […] it fitted […] [the diagnosis] helps me to understand my daughter […] [I can] read and find out how to help her[…] find resources to help her go forward.”

Both DENISE and Debbie share an initial aversive reaction to the BPD diagnosis; however, this was followed by some positive gains in terms of situating the young people's difficulties as something explainable and offering a knowledge base, from which to seek guidance, and all participants referred to this positive sense‐making.

Nevertheless, these benefits were overwhelmingly clouded in the interviews by a series of negative consequences contained within an understanding of BPD. Firstly, all young people expressed a view of themselves as divergent from normal, and in some way defective, as a direct result of the diagnosis itself, illustrated by SARAH:

Sarah “If you say ‘personality disorder’ you immediately think ‘oh god that's really bad’, because your personality is quite a big thing […] I see it that I'm ruined, I'm not ever going to be normal, that I'm not normal. It's an actual disorder of my personality, and I find that really hard.”

As illustrated by SARAH, the inclusion of the words ‘personality disorder’ in the BPD label was negatively loaded and resulted in explicit derogatory connotations and judgements about herself as ‘not normal’, or ‘ruined’. The language chosen by SARAH is powerful and emphasises the weight of this judgement for her, that to mark her personality out as ‘disordered’ meant that part of her self was spoiled and irreparably damaged. Secondly, this sense of defectiveness or difference was worsened by a shared view that BPD is permanent, as illustrated by Katherine:

Katherine “I mean I hope [KATIE] will get to a point where she can live life more fully than she is now[…] being able to be a bit more independent than she is now, being able to cope in different situations. But I think we have kind of accepted that it is you know, it probably is a lifelong issue.”

As depicted by Katherine, the understanding of the BPD diagnosis that these participants are discussing is a view of being permanently and fundamentally different, and at least for these participants, this contributed to the diagnosis being experienced as threatening.

This sense of threat was highlighted in the subtle ways that language was used throughout. All young people continually shifted perspective between viewing BPD as separate and external to them (e.g. PAUL: *‘I just thought it's another disorder; it's not going to change who I am’*), to viewing BPD as located within them (PAUL: *‘I dunno, it's just, because that's my primary disorder, I just, I dunno, that's just who I am’*). This shifting language highlighted the difficulty these young people were facing in perhaps not wanting, or being able, to truly associate themselves with the diagnosis, at least in part due to what this means about them. Paul also raises an important issue of the BPD diagnosis being one of many he has received over time, an experience which is common in those diagnosed with BPD and which may also contribute to the difficulty experienced in fully relating to it.

Some divergence of perspective was observed across the two participant groups, as well as within the mothers' accounts. Mothers were more consistent about how they viewed the BPD diagnosis but differed in terms of whether they viewed this as separate to, or within, their child. Hollie and Nicola adopted the stance of the BPD diagnosis being separate from their child (e.g. Hollie: *‘I think it's just a diagnosis. It's not who [son] is. It's just a diagnosis of what he's got’*). Debbie and Phoebe adopted a contrasting stance of BPD being located within their child, as depicted by Phoebe:

Phoebe “It was just a fact that this was [PAUL] now and it was more getting to grips with the new [PAUL], because the [PAUL] I'd had for the past 14 years was gone, I'd accepted that, it was like I had to grieve and accept the new [PAUL], because he is a completely different kid now.”

For Phoebe, a process of accepting the perceived permanent nature of the disorder seemed to have facilitated a stronger connection with the diagnosis and her child.

The subtheme ***‘Worrying that outsiders might judge, and insiders might despair’*** illustrates how the perceived negative connotations associated with the BPD diagnosis extended to views related to what others might think. Despite not always explicitly describing their views and experiences as stigma related, all participants spoke about their fears related to the possibility that sharing their/their child's BPD diagnosis could incite negative reactions and judgements from others. For example, KATIE spoke about her fears that others *‘might judge [her]’* if they found out that she had BPD, JOE stated that when he has told people about his BPD *‘they look at you differently’*, and DENISE stated that she doesn't want to share her BPD diagnosis with others ‘*because I don't want them to get scared and go, leave*.’ Debbie illustrates how mothers also viewed disclosures as risky ‘*I think sometimes in the wrong hands it can be treated in the wrong way […] when she goes off to work or places like that there may be a stigmatisation attached to it.’*

Some of the mothers provided a deeper insight into the stereotypical views that may fuel the apparent stigma associated with BPD that did not appear to be available to the young people, for example:

Phoebe “I think there is some sort of fear as to how he will react […] That he will hit something, that he will sort of switch, but they don't know, I mean [PAUL] doesn't switch emotions like that […] it isn't just going to happen because you've said something he doesn't like… [other people think that they] are dangerous, that they are angry, and that they can cause serious harm.”

In summary, the BPD diagnosis is described as providing some useful insights in terms of providing an explanation for their (or their child's) behaviour, but this is weighted against significant consequences, as the diagnosis itself was seen to indicate long‐term damage and even potential danger. As a result of these powerful negative connotations, the BPD diagnosis had become the elephant in the room, which participants were cautious about discussing.

## DISCUSSION

It is evident from Theme 1 that participants described a clear pattern of experience, reflected in the accounts of both young people and parents. At the core of this pattern is the intensity and unpredictability of the young person's emotional distress, and the accompanying felt pressure on parents to keep them safe. It seems reasonable to note that there is a shared experience here, irrespective of whether BPD is the best way of naming and accounting for that commonality. For both parents and young people, there is also a strong sense of unmet need: the distress and pressure make ‘normal life’ more difficult than it should be. Future research must seek to better understand these unmet needs and work to generate targeted solutions to support families to live alongside these difficulties.

In Theme 2, we have underlined a stark ‘cost–benefit’ divide in the consequences of the BPD diagnosis for our participants. Participants reported that the diagnosis provided them with some insight into the nature of the difficulties experienced by the young person and presumably *some* access to care, given the context of recruitment. However, participants also described how the diagnosis was very difficult to accept. For some, the difficulty of accepting a diagnosis was because it came with the connotation that the problem is integral to, and situated within, the young person themselves. This is not surprising, because that is what ‘personality’ is usually taken to indicate. Others worked hard to think of the problem as one that was distinct from their child, but all participants shared some worries about how others might avoid or mistreat the young person due to common conceptions about the meaning of ‘personality disorder’, and particularly the misplaced implication that the young person might be a danger to others.

These kinds of concerns about the potential psychosocial harms associated with the diagnosis align with several of the issues raised by clinicians who are worried about the validity and utility of the BPD diagnosis (Hartley et al., 2022). A key point made by Hartley et al.'s critique is that BPD itself is not necessarily the only route for young people to access information and intervention – and in fact, they argue that there is little evidence that the diagnosis *is* a good route for accessing these things. Given that the NICE‐recommended intervention is dialectical behaviour therapy (DBT), that not all CAMHS services can deliver this, and that access to DBT itself often involves meeting quite stringent pre‐treatment criteria, it is likely that many young people with a BPD diagnosis do not access the intervention, which is currently recommended for them.

Ultimately, the current study illustrates how the BPD diagnosis may be experienced as harmful, supporting views that change is needed. At minimum, care and consideration needs to be given to ensuring firstly that communication about the diagnosis is not avoided, and secondly to *how* the BPD diagnosis is communicated, so that this can be done in a way that dispels important misconceptions, and fosters hope not fear. If we cannot voice open and informed conversations about BPD in the clinic room with young people and families, how can we expect to begin to shift how BPD is perceived outside of the clinic room? What is perhaps more worrying, is that the stigma and misconceptions associated with a BPD diagnosis identified in this paper are not new issues and have been highlighted repeatedly (Chanen & McCutcheon, 2008; Shepherd et al., 2017; Spodenkiewicz et al. 2013). Our findings emphasise the fact that there has been little change, indicating that a focus on shifting perceptions may be futile, and supporting the view that a broader change is needed in how we conceptualise difficulties currently labelled as BPD.

It is beyond the scope of this paper to develop a case for what that conceptualisation should look like, but the wider literature provides several candidates, for example, Johnstone et al. (2018) introduced the Power Threat Meaning Framework as an optional alternative perspective to diagnostic labels in general, with a focus on adopting a narrative‐based approach to understanding distress, and Hill et al. (2023) propose a theoretical model based on social domain disorganisation. Future research efforts may be better placed at shifting gears, moving away from further investigation of the controversy surrounding the BPD diagnosis in general, and beginning to explore alternative conceptualisations. The principles of co‐production must be a priority at every level in working towards innovation.

### Strengths and limitations

Exploring the experiences of young people and mothers from their own perspectives is the key strength of the current study, as this gave voice to the individuals at the centre of the broader debate, allowing the issues that are important to them to be heard.

The multi‐perspectival design posed some challenges in terms of recruitment and ethical considerations, as well as in relation to ensuring both groups were adequately represented and reported. However, despite these nuances, the design decision reflects a key strength of the current paper providing a rich new insight into what it is like for families to live with the emotional distress and unpredictability associated with difficulties labelled as BPD, and illuminating how family relationships and roles are affected. The multi‐perspectival design also allowed the study to extend its reach and confidence in findings, as key issues are evidenced from more than one perspective, providing an opportunity for triangulation that is in‐built within the research design.

## CONCLUSION

The current paper offers a novel and powerful insight into what it is like for families to live alongside BPD, highlighting issues associated with how roles and relationships are affected, gaps in support from services, and problems associated with how the label itself is experienced. Ultimately, it is not clear to us that the concept of ‘personality disorder’ itself adds anything substantive to young people's and families' ability to understand what is distinctive about the young person's experiences and needs, or that it does so in a way which fosters hope for the young person's future. None of the nine dimensions of BPD requires a grounding in ‘personality’ in order to make sense of a difficulty or to be understood as something that may be difficult to change.

It is not a question of changing the ‘language’ here, but of providing a *conceptualisation* of the difficulties, which people experience that does not also import additional harms, and future research efforts should begin to shift attention towards re‐conceptualisation.

## AUTHOR CONTRIBUTIONS

**L. Marriner:** Conceptualization; methodology; writing – original draft; writing – review and editing; project administration; formal analysis; investigation; validation. **M. Larkin:** Conceptualization; methodology; validation; supervision; resources; writing – review and editing; writing – original draft; formal analysis. **G. Urquhart Law:** Conceptualization; validation; supervision; resources; writing ‐ original draft; writing ‐ review and editing; formal analysis. **S. Kaur Bhogal:** Conceptualization; supervision; resources.

## FUNDING INFORMATION

A small research bursary of £250 was provided by the University of Birmingham for research costs. There was no external funding.

## CONFLICT OF INTEREST STATEMENT

The authors have declared that they have no competing or potential conflicts of interest.

## ETHICS STATEMENT

IRAS ID: 237975, Sponsor Ref (University of Birmingham): RG_17‐208, REC approval: Black Country REC, REC reference: 18/WM/0063, Approval date: 03/04/2018.

## Data Availability

Due to confidentiality reasons and ethical permissions, data are not available.
